# rVSV-ZEBOV vaccination in people with pre-existing immunity to Ebolavirus: an open-label safety and immunogenicity study in Guinean communities affected by Ebola virus disease (l’essai *proches*)

**DOI:** 10.1186/s12916-024-03726-z

**Published:** 2024-11-07

**Authors:** Conall H. Watson, Pierre-Stéphane Gsell, Yper Hall, Anton Camacho, Ximena Riveros, Godwin Enwere, Andrea Vicari, Séverine Danmadji Nadlaou, Alhassane Toure, Ismaila M. Sani, Abdourahamane Diallo, Cece Kolie, Sophie Duraffour, Kékoura Ifono, Andre Maomou, Kassie Dore, Honora A. Djidonou, Aminata Bagayoko, Philos P. Damey, Mabetty Nancy Camara, Fatoumata Battouly Diallo, Fofana Thierno Oumar, Kalidou Toure, Mohamed Lamine Diaby, Lansana Sylla, Doussou Conde, Ibrahima Lansana Kaba, Tom Tipton, Rosalind M. Eggo, Michael Marks, Chrissy H. Roberts, Thomas Strecker, Stephan Günther, Sakoba Keita, W. John Edmunds, Miles W. Carroll, Ana Maria Henao-Restrepo

**Affiliations:** 1 UK Health Security Agency, London, UK; 2 World Health Organization, Geneva, Switzerland; 3 University of Oxford, Oxford, UK; 4 London School of Hygiene and Tropical Medicine, London, UK; 5 Bernhard-Nocht-Institut Für Tropenmedizin, Hamburg, Germany; 6 Philips University, Marburg, Germany; 7 Epicentre, Paris, France; 8 Ministry of Health, Conakry, Guinea

**Keywords:** Ebola virus disease, Ebola, Vaccine, RVSV-ZEBOV, Safety, Immunogenicity, Cohort study

## Abstract

**Background:**

Zaire Ebolavirus disease (EVD) outbreaks can be controlled using rVSV-ZEBOV vaccination and other public health measures. People in high-risk areas may have pre-existing antibodies from asymptomatic Ebolavirus exposure that might affect response to rVSV-ZEBOV. Therefore, we assessed the impact pre-existing immunity had on post-vaccination IgG titre, virus neutralisation, and reactogenicity following vaccination.

**Methods:**

In this prospective cohort study, 2115 consenting close contacts (“*proches*”) of EVD survivors were recruited. *Proches* were vaccinated with rVSV-ZEBOV and followed up for 28 days for safety and immunogenicity. Anti-GP IgG titre at baseline and day 28 was assessed by ELISA. Samples from a representative subset were evaluated using live virus neutralisation.

**Results:**

Ten percent were seropositive at baseline. At day 28, IgG in baseline seronegative (GMT 0.106 IU/ml, 95% CI: 0.100 to 0.113) and seropositive (GMT 0.237 IU/ml, 0.210 to 0.267) participants significantly increased from baseline (both *p* < 0.0001). There was strong correlation between antibody titres and virus neutralisation in day 28 samples (Spearman’s rho 0.75). Vaccinees with baseline IgG antibodies against Zaire Ebolavirus had similar safety profiles to those without detectable antibodies (63.6% vs 66.1% adults experienced any adverse event; 49.1% vs 60.9% in children), with almost all adverse events graded as mild. No serious adverse events were attributed to vaccination. No EVD survivors tested positive for Ebolavirus by RT-PCR.

**Conclusions:**

These data add further evidence of rVSV-ZEBOV safety and immunogenicity, including in people with pre-existing antibodies from suspected natural ZEBOV infection whose state does not blunt rVSV-ZEBOV immune response. Pre-vaccination serological screening is not required.

**Supplementary Information:**

The online version contains supplementary material available at 10.1186/s12916-024-03726-z.

## Background

Ebolavirus (EBOV) has caused high mortality and morbidity in outbreaks of Ebola virus disease (EVD) across equatorial Africa [1, 2]. Sustained person-to-person transmission may arise following single zoonotic transmission events and lead to large outbreaks [3–5].

EVD control methods include contact tracing, case isolation, health education, and safe burial, with appropriate community engagement [6]. Since 2016, outbreak response also includes the use of a safe and highly efficacious attenuated replication-competent vesicular stomatitis virus-derived vaccine expressing the surface glycoprotein (GP) of Zaire ebolavirus ZEBOV (rVSV-ZEBOV) [7, 8].

Previous studies report that rVSV-ZEBOV is safe, well tolerated [7–13], and has been approved by US and European regulators as well as national regulatory authorities in ten African countries. Serology studies from central Africa reported that in villages experiencing an outbreak and in areas at risk of zoonotic transmission mean population seroprevalence will range from 2 to 29%, as measured by the detection of immunoglobulin G (IgG) to EBOV-GP in clinical samples [14–18]. Studies in post-outbreak communities in West Africa have similarly found undiagnosed seropositive contacts, referred to as asymptomatic cases, though a level of caution needs to be applied to studies in which antibody analysis is not supported by additional assessments such as virus neutralisation.

The impact of pre-existing antibodies to EVD on rVSV-ZEBOV safety and immunogenicity is unknown. Any sign of blunted immunogenicity or safety concerns in previously infected individuals would warrant further investigation. Therefore, we undertook an open-label rVSV-ZEBOV vaccination cohort study, enrolling contacts of EVD cases who survived and for which ring vaccination was not previously implemented during the 2013–2016 outbreak [19–21].

This study’s primary objective was to measure the IgG response to EBOV GP at 28 days post-vaccination by baseline IgG status; secondary objectives were comparing the rates of serious adverse events (SAEs) and adverse events (AEs) by baseline serostatus; and to explore any association between participants’ baseline serostatus and linked-survivor-related factors.

## Methods

### Setting

The study was conducted in Basse-Guinée region, Guinea. The first volunteer was recruited on 25 May 2016 and follow-up of the final participant completed on 21 Sep 2016.

### Design

Participants were contacts of EVD survivors whose onset of symptoms occurred within 15 months prior to the start of the study. The study was a prospective cohort study examining IgG antibodies by ELISA serology at baseline and at day 28 following rVSV-ZEBOV vaccination. It also documented post-vaccination adverse events at days 3, 14, and 28, comparing those with raised baseline antibodies against those without. Survivors were tested using RT-PCR and were not prospectively vaccinated. Baseline and day 28 EBOV GP IgG titres were also assessed for association with survivor-related factors, such as time since survivor EVD onset.

### Study population

In May 2016, we identified contacts of adult or adolescent, PCR confirmed, EVD survivors who had not been previously included in the rVSV-ZEBOV ring vaccination *Ebola ca suffit trial* [7]. These contacts were assumed to be at a low but not zero risk of EVD due to potential persistence of viable EBOV in the body fluids of EVD survivors [14, 20, 22, 23]. Survivors were identified from national surveillance records. Survivor support organisations were also used as an information source to verify data against these records. Survivors’ communities of residence were cross-referenced against the records of the Ebola Ça Suffit ring trial [7], to exclude survivors whose contacts were already included in the trial.

A revised definition of contacts of EVD survivors because of their socio-epidemiological status (“*les proches*”) was developed. It included household contacts (shared sleeping building or shared cooking pot), sexual contacts, and second tier of people who would be at risk of transmission should a close contact develop EVD as a result of a post-recovery transmission event, e.g. neighbours of survivors and household contacts of a sexual contact of a survivor.

*Proches* were identified by trained field epidemiologists. They were eligible for inclusion if they were 6 years of age or older and did not meet any exclusion criteria, namely: history of EVD (self-report), history of vaccination with any EBOV vaccine, verbally declared pregnancy, or planning to conceive within 2 months of vaccination (pregnancy tests were offered but not required), breast-feeding, history of having received investigational research agents in the previous 28 days, clinically important immunodeficiency condition (e.g. HIV/AIDS), history of anaphylaxis to a vaccine or vaccine component, or a severe illness that makes the person bed-bound or requiring hospitalisation at the time of the vaccination.

### Procedures

Consenting vaccinees received a single dose of at least 2 × 10^7^ plaque forming units of the rVSV-ZEBOV vaccine (donated by Merck Sharp & Dohme, Kenilworth, NJ, USA) intramuscular to a deltoid. From each vaccinee, immediately prior to vaccination and on day 28 (± 3 days) post-vaccination, up to 10 ml of venous blood for serology was collected in a sterile serum separator tube by a trained person using standard techniques. Participants were observed for 30 min post-vaccination for immediate adverse reactions. Follow-up for adverse events was done on days 3, 14, and 28 post-vaccination by safety monitoring teams, with a window of ± 1, 2, and 3 days respectively.

Consenting EVD survivors provided a 10 ml venous blood for reverse-transcriptase polymerase chain reaction (RT-PCR) in the study laboratory in Conakry, Guinea. Female survivors were invited to provide a vaginal swab, and menstrual blood or breastmilk sample if applicable; male survivors were invited to provide a semen sample. All were tested using an RT-PCR.

Samples were transferred to Europe for analysis under a signed Material Transfer Agreement issued by the Ministre Sante Hygiene Publique, Guinée.

### Data collection

Data was collected on encrypted Android tablets using OpenDataKit (ODK) Collect on forms designed in XLSform. Uploaded data was stored in ODK Aggregate on an encrypted server. QR barcodes were used to link participants’ records and laboratory samples and for staff digital signatures. GPS data was collected on participant locations to aid follow-up.

Baseline demographic data and contact information were collected from all participants. Survivors were asked about possible EVD sequelae. Details of specified adverse events were solicited at each post-vaccination follow-up visit, with unsolicited events also documented on days 3 and 14. Participants were provided with a contact number on their participant enrolment card if they wanted to contact the study team between visits to notify or get advice on any possible adverse events.

To reduce the risk of important response biases, data collectors were trained in information solicitation methods by experienced HIV/AIDS teams. Interviews were conducted away from communal settings to increase privacy. Participants missing from safety monitoring visits were actively traced by the field team to reduce loss to follow-up.

### Laboratory methods

#### EBOV GP ELISA

Anti-EBOV GP IgG was determined by ELISA using previously published methods [24, 25] with a trimerised Zaire strain GP antigen, aa 1–649 of GenBank AHX24649.1 (GIN/2014/Makona-Kissidougou-C15). Results were standardised by inclusion of a reference curve for which the reference material was a plasma pool from three EVD survivors, calibrated against WHO Reference Reagent (NIBSC 15/220) [26] (Anti-GP titre = 0.761 ± 0.006 IU/ml (mean ± SEM, *n* = 13)). Negative control plasma pool was obtained from three Guinean volunteers with no prior infection with EVD or contact with an EVD case-patient. Matched serum pairs were tested in triplicate. Samples without a matching pair were not tested. OD was interpolated from the reference serum curve. In the absence of well-defined thresholds for immunity or past infection, we set a serological threshold based on the negative control. The threshold for seropositivity was determined as 0.0429 IU/ml, three standard deviations above the negative control geometric mean (*n* = 91).

### EBOV Zaire neutralisation assay

Fifty paired samples were selected based on having a low, medium, or high baseline anti-GP IgG titres for analysis using a live EBOV neutralisation assay at the Institute of Virology BSL4 laboratory, Philipps University of Marburg, Germany [27]. Based on previous studies, the seropositivity threshold for neutralisation assays was defined as ≥ 1:8 geometric mean titre (GMT).

### Statistical analysis

To recruit 50 baseline-seropositive participants, a sample size of 1000 ≤ *n* ≤ 2000 was estimated to be required if pre-existing seropositivity was consistent with serosurveillance in other Ebola-experienced areas.

Descriptive analyses and multivariable regression were performed in R (3.5) [28]. For multivariable regression where relevant variables were missing, records were omitted from that analysis. Serological data was compared by paired *t*-test or Mann–Whitney test. Correlation between ELISA and neutralisation assays was compared by Spearman rank correlation coefficient. We set statistical significance level at 5%.

## Results

**Fig. 1:**
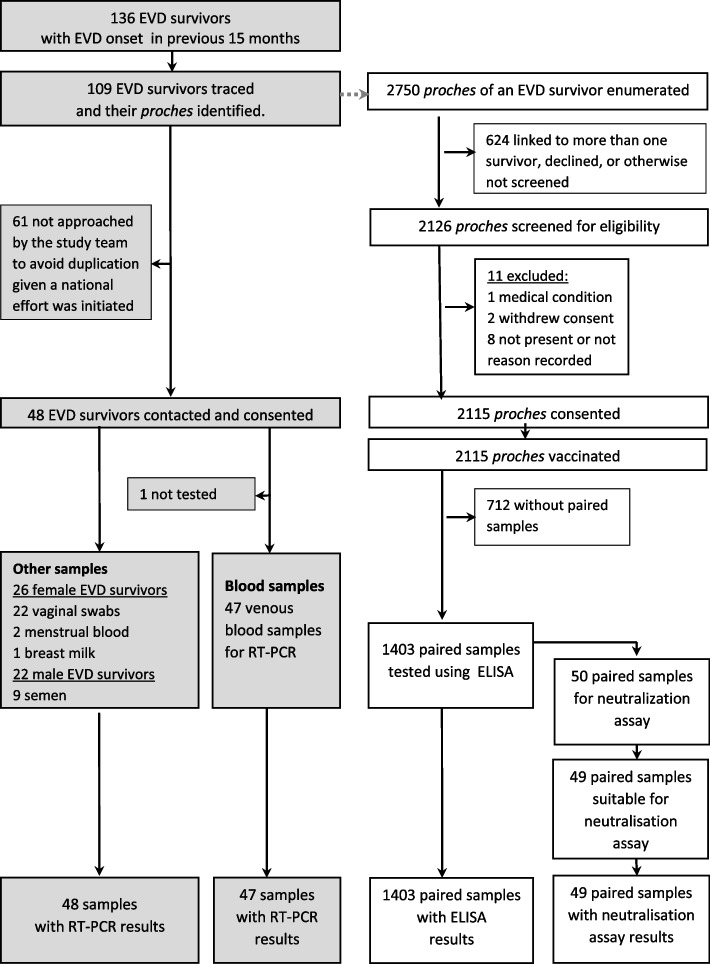
Study flowchart

We identified 136 EVD survivors whose contacts (*proches*) were unvaccinated. For these survivors, the date of discharge from Ebola treatment unit ranged from 16 Feb to 7 Oct 2015 (median 22 March 2015). Of these, 80.1% (109/136) were located. Only 48 were contacted and all gave their consent for an interview and for sample collection. The remaining were not approached by the study team because a national effort with similar objectives was initiated, and duplication was deemed not desirable. In total, 2750 *proches* of 109 EVD survivors were identified. Amongst them 77.3% (2126/2750) were eligible for our study, and 2115 were enrolled and vaccinated following consent (Fig. 1). The median number of screened and vaccinated *proches* per EVD survivor was 12 (range 1 to 100, IQR 7 to 21). Of the 0.5% (11/2126) not then vaccinated, eight were either not present at the time of vaccination or did not have a reason for non-vaccination status, two withdrew consent, and one had a medical condition. Venous samples from both day 0 and day 28 were provided by 66.3% (1403/2115) of the vaccinees.

### Humoral immunogenicity following rVSV-ZEBOV vaccination

We tested the 1403 matched (day 0 and 28) *proches’* samples by ELISA and found that 228 (16.3%) were defined as seropositive at baseline (day 0).

Logistic regression analysis of potential factors associated with baseline seropositivity did not find an association with sex, age group, or time since survivor discharge from the Ebola treatment unit (ETU) (Additional file: Table S1).

**Fig. 2:**
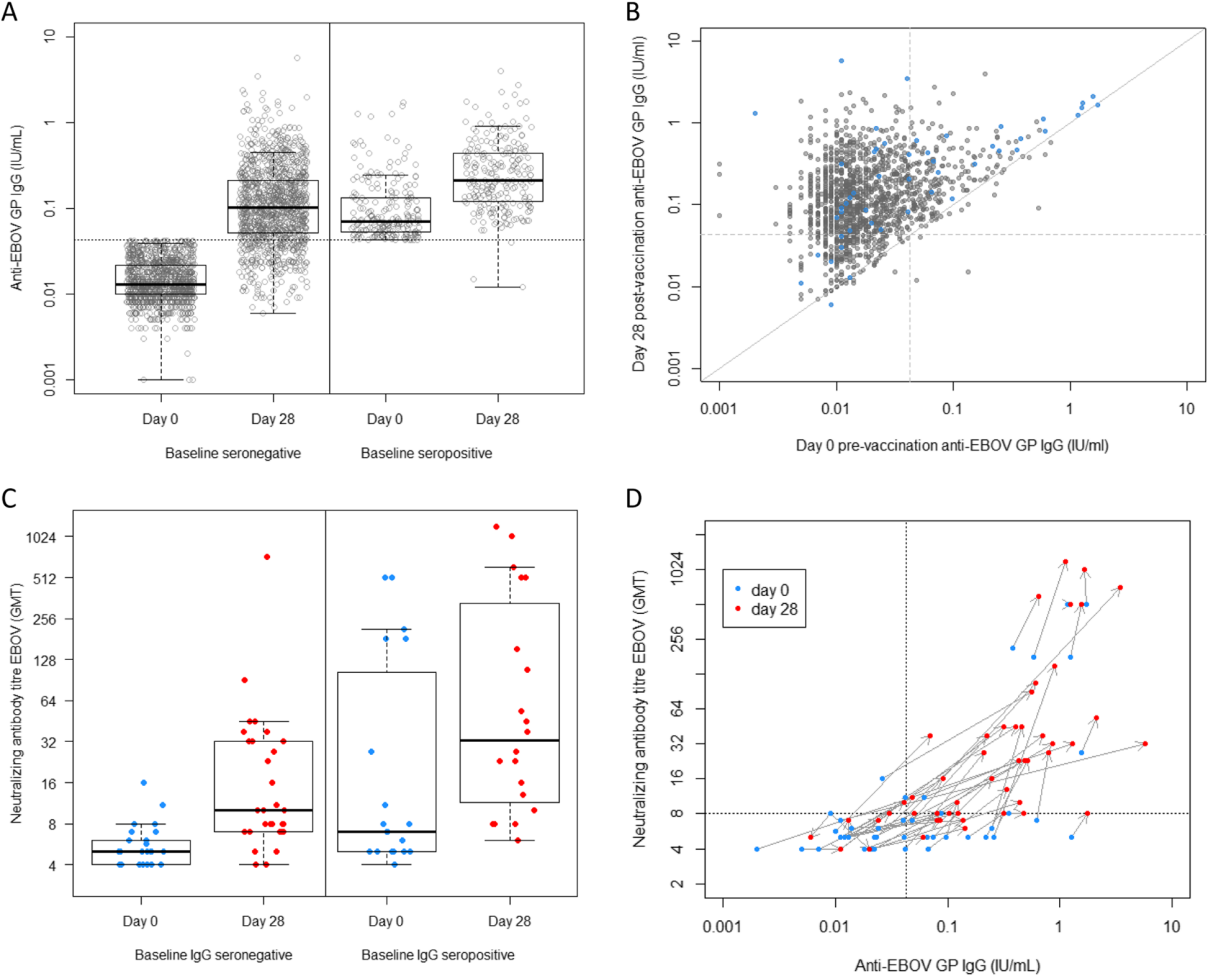
**A** Pre- and 28-day post-vaccination anti-EBOV IgG ELISA titres, stratified by baseline seropositivity status. Box indicates median and interquartile range (IQR), whiskers 1.5*IQR. **B** Day 28 post-vaccination anti-EBOV IgG ELISA titres against pre-vaccination titres. Blue points indicate samples selected on ELISA for neutralisation assay and tested successfully. **C** Pre- and 28-day post-vaccination Ebolavirus neutralisation titres, stratified by baseline anti-EBOV IgG ELISA titres in the subset. Box indicates median and interquartile range, whiskers 1.5*IQR. **D** Neutralisation titres before and 28 days after vaccination in a subset of samples tested by IgG ELISA

Of the 1175 baseline seronegative participants, 955 (81.3%, 95% CI 78.9 to 83.4%) were seropositive by ELISA on day 28 (Fig. 2A and B). Three (1.3%) of 228 initially seropositive vaccinees were found seronegative at day 28. This could be due to waning immunity in weakly positive samples or assay variability. There were statistically significant increases in IgG GMT at day 28 for both those seropositive and seronegative at baseline (Table 1). The day 28 GMT was higher for baseline seropositive participants compared to the day 28 GMT of baseline seronegative participants (*p* value for difference < 0.0001).

**Table 1:** Participant demographics and IgG titres by baseline serostatus

	*Proches* vaccinees (*n* = 2115, of whom 1403 had paired serum for analysis)			EVD survivors (*n* = 48)
Serostatus	Seronegative at baseline (day 0)	Seropositive at baseline (day 0)	Not tested (no paired sample)	Not applicable
Number (% of those tested)	1175 (83.7%)	228 (16.3%)	712	48
Female sex (%)	440 (37.4%)	81 (35.5%)	239 (33.6%)	26 (54.2%)
Median age (IQR)	28 (15 to 46)	30 (18 to 46)	26 (18 to 40)	32 (22 to 40)
Age range	6 to 99	6 to 85	6 to 90	15 to 66
Children age 6–17 years (% of those tested)	338 (86.0%)	55 (14.0%)	172	4
Adults 18 years + (% of those tested)	837 (82.9%)	173 (17.1%)	540	44
IgG geometric mean titre (GMT) (95% confidence interval (CI), *p* value for difference from baseline)				
Day 0 pre-vaccination	0.0142 (0.0138 to 0.0146)	0.0927 (0.0838 to 0.103)	N/A	N/A
Day 28 post-vaccination	0.106 (0.100 to 0.113, *p* < 0.0001)^*^	0.237 (0.210 to 0.267, *p* < 0.0001)^*^	N/A	N/A
Seroconversion at day 28 (%; 95% CI)				
Children	308/338 (91.1%, 87.6 to 93.7%)^†^	N/A	N/A	N/A
Adults	647/837 (77.3%, 74.3 to 80.0%)^†^	N/A	N/A	N/A
GMT (95% CI)				
Children’s baseline	0.0130 (0.0122 to 0.0138)	0.096 (0.077 to 0.120)	N/A	N/A
Children’s day 28	0.167 (0.149 to 0.187)^‡^	0.338 (0.266 to 0.429)	N/A	N/A
Adults’ baseline	0.0147 (0.0142 to 0.0152)	0.092 (0.082 to 0.103)	N/A	N/A
Adults’ day 28	0.089 (0.083 to 0.095)^‡^	0.211 (0.185 to 0.242)	N/A	N/A

^*,†,‡^The *p* value for difference between these paired groups was < 0.0001

Examining IgG titres of those who were seronegative at baseline, we found that children showed 91.1% seroconversion rate (308 of 338, 95% CI 87.6 to 93.7%) compared with adults who showed a 77.3% seroconversion rate by day 28 (647 of 837, 95% CI 74.3 to 80.0%). Children also had higher day 28 IgG GMT of 0.167 (95% CI 0.149 to 0.187) compared to adults GMT of 0.089 (95% CI 0.083 to 0.095, *p* < 0.0001). Amongst those classed as seropositive at baseline, we found that children attained higher day 28 titres (GMT 0.338, 95% CI 0.266 to 0.429) than adults (GMT 0.211, 95% CI 0.185 to 0.242, *p* = 0.003).

We next sought to validate these ELISA results using live virus neutralisation assays on a subset of 50 matched day 0 and 28 samples, 100 samples in total. These samples were identified as high, medium, or low responder. We found that day 0 neutralisation GMT was 8.5 (95% CI 5.9 to 12.2), rising to 25.1 (95% CI 16.1 to 39.2) at day 28, with proportion considered positive rising from 13/49 (26.5%) to 39/49 (79.6%). Moderate correlation with IgG was seen in baseline neutralisation assay titres (Spearman’s rho = 0.50; *p* = 0.002 on 47 degrees of freedom (df)). Correlation was stronger between day 28 assays (rho = 0.75; *p* < 0.0001 on 47 df) (Fig. 2D).

### Vaccine safety

We also investigated adverse events and stratified this by serostatus at baseline and age group (Table 2). Although the immunogenicity analysis was restricted to vaccinees providing paired serological samples, safety data is also presented for those who did not provide a day 28 serum sample. Prevalence was similar in both serogroups and those untested. Of the 62% (1317/2215) reporting any adverse event, 99% (1038/1317) reported only mild events. The most common adverse event in adults and children was headache (35.1% (742/2115)), followed by fatigue (23.7% (501/2115)) and muscle pain (14.6% (308/2115)) (Table 2). Most were short duration: median 2 days (interquartile range 1–3). Participants most commonly reported adverse events at the day 3 follow-up visit. The most common unsolicited adverse events in adults were shivers/chills (3.6% (56/1550)), dizziness (2.3% (36/1550)), and abdominal pain (1.2% (18/1550)); amongst children the more frequent adverse events include abdominal pain (4.2% (24/565)) and shivers/chills (2.1% (12/565)) (Table 2).

**Table 2:** Number of adults and children who experienced specified and unsolicited adverse events during 28 days from vaccination, by pre-vaccination serostatus

	**Adults**			**Children**		
Serostatus	Seronegative at baseline *n* = 837	Seropositive at baseline *n* = 173	Not tested (no paired sample) *n* = 540	Seronegative at baseline *n* = 338	Seropositive at baseline *n* = 55	Not tested (no paired sample) *n* = 172
Specified adverse events						
Any adverse event	553 (66.1%)	110 (63.6%)	321 (59.4%)	206 (60.9%)	27 (49.1%)	100 (58.1%)
Diarrhoea	15 (1.8%)	1 (0.6%)	13 (2.4%)	6 (1.8%)	1 (1.8%)	1 (0.6%)
Fatigue	220 (26.3%)	44 (25.4%)	150 (27.8%)	52 (15.4%)	6 (10.9%)	29 (16.9%)
Headache	306 (36.6%)	60 (34.7%)	160 (29.6%)	136 (40.2%)	17 (30.9%)	63 (36.6%)
Injection pain	27 (3.2%)	4 (2.3%)	21 (3.9%)	23 (6.8%)	–	15 (8.7%)
Joint pain	134 (16%)	35 (20.2%)	86 (15.9%)	21 (6.2%)	2 (3.6%)	10 (5.8%)
Muscle pain	155 (18.5%)	24 (13.9%)	69 (12.8%)	32 (9.5%)	5 (9.1%)	23 (13.4%)
Vomiting	12 (1.4%)	–	6 (1.1%)	13 (3.8%)	–	3 (1.7%)
Other (unsolicited) adverse events comprising:	111 (13.3%)	20 (11.6%)	62 (11.5%)	47 (13.9%)	5 (9.1%)	19 (11%)
Abdominal distension	1 (0.1%)	–	–	–	–	–
Abdominal pain	11 (1.3%)	–	7 (1.3%)	17 (5%)	1 (1.8%)	6 (3.5%)
Blurred vision	2 (0.2%)	–	2 (0.4%)	–	–	–
Buccal inflammation	–	–	1 (0.2%)	–	–	–
Constipation	1 (0.1%)	–	–	–	–	–
Cough	4 (0.5%)	–	1 (0.2%)	2 (0.6%)	–	2 (1.2%)
Dizziness	20 (2.4%)	5 (2.9%)	11 (2%)	4 (1.2%)	–	1 (0.6%)
Epigastric pain	6 (0.7%)	1 (0.6%)	2 (0.4%)	1 (0.3%)	–	–
Fever	4 (0.5%)	–	–	1 (0.3%)	–	1 (0.6%)
Hyperhidrosis	2 (0.2%)	–	–	–	–	–
Insomnia	1 (0.1%)	–	–	–	–	–
Loss of appetite	7 (0.8%)	–	6 (1.1%)	–	–	–
Lower back pain	14 (1.7%)	7 (4%)	11 (2%)	1 (0.3%)	–	–
Nausea	2 (0.2%)	–	–	–	–	–
Polyuria	1 (0.1%)	–	–	–	–	–
Pruritus	6 (0.7%)	3 (1.7%)	3 (0.6%)	–	–	–
Shivering/chills	36 (4.3%)	4 (2.3%)	16 (3%)	8 (2.4%)	2 (3.6%)	2 (1.2%)

Eight participants reported moderate adverse events (most commonly fatigue or muscle pain) and one reported severe fatigue (Table 3). No moderate or severe adverse events were reported in those known to be seropositive at day 0.

**Table 3:** Moderate and severe adverse events

Participant	Baseline serostatus	Adverse event	Day of onset post-vaccination	Duration (days)^b^
7 year old girl	Negative	Fatigue	28	2
		Muscle pain	28	2
18 year old man	Negative	Headache	1	1
		Joint pain	1	1
		Muscle pain	1	1
18 year old woman	Negative	Nausea	1	1
19 year old man	Unknown	Dizziness	1	≥ 3, ≤ 14
		Loss of appetite	1	≥ 3, ≤ 14
		Buccal inflammation	1	13
		Itching	1	13
21 year old man	Unknown	Fatigue	0	3
35 year old man	Negative	Joint pain	1	≥ 3, ≤ 14
		Muscle pain	1	4
42 year old man	Unknown	Fatigue	1	2
		Muscle pain	1	2
		Lower back pain	1	2
49 year old man	Unknown	Fatigue^a^	24	≥ 4
		Headache	24	≥ 4
63 year old man	Unknown	Fatigue	1	2

^a^Denotes reported as severe. All other were moderate

^b^Where precise duration is not known, ranges are given from follow-up intervals

Association between any adverse event and baseline serostatus was assessed by logistic regression, adjusting for age group (adult/child) and sex and interaction between these factors (Additional file: Table S2). No association was observable for baseline serological status; adult females appeared more likely to report adverse events than other groups.

In an exploratory Poisson regression of adverse event count (across solicited and unsolicited AEs, Additional file: Table S3), there was weak evidence of association between baseline seropositivity and lower count of adverse events after adjusting for age and sex. No interactions were identified in sensitivity analysis. Results did not change under robust Poisson regression.

### EVD survivors

All 48 consented survivors completed a questionnaire, of whom 14 (29.2%) reported at least one health problem since discharge. Eye problems were most common (10, 20.8%). Sequelae were reported across multiple organ systems including neurological signs, joint problems, and abdominal symptoms. From the 128 body fluid samples tested (see Fig. 1), none tested positive for Ebolavirus by RT-PCR.

## Discussion

This cohort study in Guinea provides additional evidence that one dose of rVSV ZEBOV GP vaccine is safe and immunogenic amongst seropositive or seronegative individuals as assessed by IgG antibody titres to Zaire ebolavirus at the time of vaccination. This supports previous work which has shown this vaccine to be safe and effective and highlights the importance of rVSV-ZEBOV in controlling future Zaire ebolavirus outbreaks [7, 29].

The finding that 16.3% of *proches*had detectable IgG to EBOV-GP is consistent with previous community/contact studies in central Africa [14–18], and warrants further investigation into the prevalence of asymptomatic cases and the mechanisms behind asymptomatic or abortive EBOV infections [30]. Researchers found ~ 15% of study participants in Guinea, Sierra Leone, and Liberia in 2018 had prior EBOV-GP immunity before vaccination compared to only 5% in neighbouring Mali [31]. Proposals for this seemingly higher level of seroprevalence include the geographical and ecological differences between these two regions and prior exposure to related and potentially non-pathogenic filoviruses, such exposures may account for some of the elevated baseline responses seen in this study [32, 33]. We did not observe an association between baseline seropositivity and age or gender, unlike a DRC study [34].

From our results, we estimated a seroconversion rate of 81.3% (95% CI 78.9 to 83.4%) in those who were seronegative at baseline, and an overall seropositivity of 84.1% (95% CI 82.1 to 86.0%) across all *proches*tested on day 28. This is within the range of other immunogenicity studies with the rVSV-ZEBOV vaccine that will have also used different assay methodology and definitions of positive outcomes [11]. Importantly, these results provide short-term data that individuals with pre-existing immunity to EBOV GP could experience an increase of antibody titres following the administration of rVSV-ZEBOV.

The strong correlation observed between neutralisation assays and IgG ELISAs following vaccination further suggests the ELISA used may be an acceptable marker of immunogenicity.

The frequency of adverse events between the serogroups was similar. No increase in adverse events reporting was identified when serogroups were further stratified into children and adults.

It was not possible to assess the effects of vaccination in prevention of infection in contacts of survivors as no EVD survivor tested positive for Ebolavirus by RT-PCR. Survivor studies have shown that RNA detection decays over time in semen at variable rates in different survivors, and, as a consequence, transmission risks decrease over time due to lower inoculum, nonviable virus, or other factors. Since 2016, ≥ 95% of the EVD cases and ≥ 95% of their contacts and contacts of contacts have been enumerated in rings and vaccinated with rVSV ZEBOV GP. Therefore, a small fraction of contacts not yet vaccinated are sparsely spread over large areas, making it difficult to further assess this question in the field. It is also unknown if vaccine induced antibodies can reach the immune-privileged sites where it is assumed the virus can remain. In 2021, one case of Ebola transmission from a person infected in the 2013–2016 epidemic occurred, outside of our vaccination area [23].

## Conclusions

Ebola remains a pressing public health concern in many sub-Saharan countries. This *proches* vaccination study suggests rVSV ZEBOV GP can be safely administered to individuals with prior asymptomatic Ebola infection (or cross-reactivity that may have caused antibody production) without requiring baseline serological results. Furthermore, existence of prior EBOV GP IgG responses from suspected natural infection does not prevent the ability of an additional dose of rVSV ZEBOV GP to increase antibody titres.

## Supplementary Information

Additional file 1: Table S1. Adjusted logistic regression analysis of risk factors for baseline seropositivity. Table S2. Association between baseline seropositivity, demographic risk factors, and the occurrence of adverse events. Table S3. Association between baseline seropositivity, demographic risk factors, and the number of adverse events in a participant.

## Data Availability

The data that support the findings of this study are not openly available due to reasons of sensitivity and are available from the corresponding author upon reasonable request.

## Abbreviations

AEs: Adverse events
df: Degree of freedom
ETU: Ebola treatment unit
EVD: Ebola virus disease
EBOV: Ebolavirus
GMT: Geometric mean titre
GP: Glycoprotein
IgG: Immunoglobulin G
ODK: OpenDataKit
RT-PCR: Reverse-transcriptase polymerase chain reaction
SAEs: Serious adverse events
WHO: World Health Organization
EVD: Zaire Ebolavirus disease
rVSV-ZEBOV: Zaire ebolavirus ZEBOV
