# Supplementary material for: rVSV-ZEBOV vaccination in people with pre-existing immunity to Ebolavirus: an open-label safety and immunogenicity study in Guinean communities affected by Ebola virus disease (l’essai proches)
Source: BMC Med. 2024 Nov 7;22:523. doi: 10.1186/s12916-024-03726-z (PMC11545826; doi:10.1186/s12916-024-03726-z)
Supplement: Supplementary file 1 — Additional file 1: Table S1. Adjusted logistic regression analysis of risk factors for baseline seropositivity. Table S2. Association between baseline seropositivity, demographic risk factors, and the occurrence of adverse events. Table S3. Association between baseline seropositivity, demographic risk factors, and the number of adverse events in a participant. [file 12916_2024_3726_MOESM1_ESM.docx]

**Additional files**

**rVSV-ZEBOV vaccination and prior Ebola immunity: an open-label safety and immunogenicity study in Guinean communities affected by Ebola virus disease (l’essai Proches)**

Table of content:

Contents

[*Table S1. Adjusted logistic regression analysis of risk factors for baseline seropositivity (n=1403).* 2](#_Toc175923453)

[*Table S2. Association between baseline seropositivity, demographic risk factors, and the occurrence of adverse events.* 2](#_Toc175923454)

[*Table S3. Association between baseline seropositivity, demographic risk factors, and the number of adverse events in a participant.* 3](#_Toc175923455)

# Table S1. Adjusted logistic regression analysis of risk factors for baseline seropositivity (n=1403).

| Risk factor for baseline seropositivity | Adjusted odds ratio (95% confidence interval) | p-value |
| --- | --- | --- |
| Adult | 1.23 (0.89 to 1.73) | 0.213 |
| Female | 0.97 (0.71 to 1.3) | 0.817 |
| Days since survivor discharged from care | 0.999 (0.996 to 1.001) | 0.209 |

# Table S2. Association between baseline seropositivity, demographic risk factors, and the occurrence of adverse events.

| Risk factor for any adverse event | Adjusted odds ratio (95% confidence interval) | p-value |
| --- | --- | --- |
| Baseline seropositive | 0.82 (0.62 to 1.11) | 0.196 |
| Adult | 1.1 (0.81 to 1.5) | 0.537 |
| Female | 0.98 (0.65 to 1.47) | 0.92 |
| Adult × female | 1.72 (1.05 to 2.82) | 0.032 |

# Table S3. Association between baseline seropositivity, demographic risk factors, and the number of adverse events in a participant.

| Risk factor for increase in count of adverse events | Adjusted odds ratio (95% confidence interval) | p-value |
| --- | --- | --- |
| Baseline seropositive | 0.88 (0.76 to 1.01) | 0.066 |
| Adult | 1.29 (1.15 to 1.45) | <0.001 |
| Female | 1.24 (1.12 to 1.37) | <0.001 |

Additional exploratory logistic regression analysis was undertaken as follows:

1. Association between day 28 IgG sero-positivity status and per participant occurrence of adverse events, after adjusting for day 0 sero-positivity status, age group and sex, with interaction (an extension of primary analysis table 5). This found no association.
2. Association between log IgG titres at day 0 and per-participant occurrence of adverse events, after adjusting for day 0 sero-status, age group and sex, with interaction (a variation of table 5). This found weak evidence of association (p=0.099) between higher titres and lower likelihood of adverse events.
